# Gene, protein, and network of male sterility in rice

**DOI:** 10.3389/fpls.2013.00092

**Published:** 2013-04-11

**Authors:** Kun Wang, Xiaojue Peng, Yanxiao Ji, Pingfang Yang, Yingguo Zhu, Shaoqing Li

**Affiliations:** ^1^State Key Laboratory of Hybrid Rice, Key Laboratory for Research and Utilization of Heterosis in Indica Rice, Ministry of Agriculture, College of Life Sciences, Wuhan UniversityWuhan, People's Republic of China; ^2^Key Laboratory of Plant Germplasm Enhancement and Speciality Agriculture, Wuhan Botanical Garden, Chinese Academy of SciencesWuhan, People's Republic of China; ^3^Key Laboratory of Molecular Biology and Gene Engineering, College of Life Science, Nanchang UniversityNanchang, People's Republic of China

**Keywords:** gene, male sterility, molecular mechanism, protein, rice

## Abstract

Rice is one of the most important model crop plants whose heterosis has been well-exploited in commercial hybrid seed production via a variety of types of male-sterile lines. Hybrid rice cultivation area is steadily expanding around the world, especially in Southern Asia. Characterization of genes and proteins related to male sterility aims to understand how and why the male sterility occurs, and which proteins are the key players for microspores abortion. Recently, a series of genes and proteins related to cytoplasmic male sterility (CMS), photoperiod-sensitive male sterility, self-incompatibility, and other types of microspores deterioration have been characterized through genetics or proteomics. Especially the latter, offers us a powerful and high throughput approach to discern the novel proteins involving in male-sterile pathways which may help us to breed artificial male-sterile system. This represents an alternative tool to meet the critical challenge of further development of hybrid rice. In this paper, we reviewed the recent developments in our understanding of male sterility in rice hybrid production across gene, protein, and integrated network levels, and also, present a perspective on the engineering of male-sterile lines for hybrid rice production.

As one of the highest yield crops, rice (*Oryza sativa*) has been used as a staple food for people over 10,000 years. Being high in carbohydrates, low in fat, and rich in protein, rice has become one of the major food supplies for the expanding world population. Today, over 50% of the global population and three quarters of the Asian population live on rice. However, facing the challenge of population explosion and reducing amount of cropland in the world, it is obvious that improve the yield of cereal crops like rice, wheat, and corn are the only way to solve this problem (Lin and Yuan, 1980; Stuber, 1994). Utilizing heterosis is an effective approach to increase crop plant grain yields, and hybrid rice has been popularized in China over the last 30 years. Hybrid rice is mainly based on the three-line and two-line male-sterile systems. Three-line male-sterile system is comprised by the male-sterile line, maintainer line and restorer line, defective nuclear-cytoplasm interaction in the male-sterile line leads to sterility. Boro II (BT), Honglian (HL), and Wild abortive (WA) are three major types of cytoplasmic male sterility (CMS) resources, and are used for the breeding of three-line hybrid rice (Lin and Yuan, 1980). The two-line hybrid rice system is based on the discovery and application of environmentally sensitive genetic male-sterile lines (EGMS), which serve as both the male-sterile lines and maintainer lines under different environmental conditions. Photoperiod-sensitive (PGMS) lines and thermo-sensitive (TGMS) lines are two major types of EGMS germplasm resources. During the past two decades, great achievements have been made in improving rice yields in China using two-line hybrid rice (Yuan, 1994). Since hybrid rice is a major approach for increasing the yield potential of rice, understanding the molecular mechanisms underlying these male sterility lines in rice is critical for improvements in rice hybrid seed production technology. Recently, genes responsible for the CMS and EGMS trait in rice have been cloned. This article focuses on recent investigations of genes and proteins that are associated with male sterility in rice and provides novel insights into male-sterile pathways of rice.

## CMS in rice

CMS is a maternally inherited phenomenon found in the plant kingdom (Young and Hanson, 1987). CMS leads to pollen abortion but does not affect female fertility and vegetative growth, and fertility can be rescued by fertility restorers (Rf). The CMS/Rf system eliminates the need for hand emasculation; therefore, it is important in commercial hybrid seed production. In most cases, the failure of pollen development in a CMS background is suggested to be associated with chimeric open reading frames (ORFs) that have arisen from unusual recombination events in mitochondria (Linke and Borner, 2005). Many mitochondrial genes that responsible for CMS can be suppressed or counteracted by the products of one or more nuclear genes known as *restorer-of-fertility* genes (Hanson and Bentolila, 2004). Thus, apart from the agronomic importance of CMS, study of the trait of CMS provides a convenient way to dissect genetic interactions between the mitochondria and the nucleus in plants. However, the pollen-specific phenotype in CMS system, and the technical infeasibility of isolating and studying mitochondria from this tissue, increases the difficulties in studies the mechanism of CMS in plants.

In rice, there are three CMS/RF systems, which are named CMS-BT, CMS-WA, and CMS-HL. They have been defined by distinct genetic and cytological features.

## CMS-BT

The BT-type rice is the most fully characterized system of CMS in rice. In BT-type CMS, the cytoplasm derived from the rice line Chinsurah BoroII causes male sterility when combined with the nucleus from the rice line Taichung 65 that carries no restorer gene (Shinjyo, 1969). In the mitochondrial genome of Chinsurah Boro II, a chimeric gene called *orf79*, located downstream of e *atp6*, which encodes a cytotoxic peptide, was confirmed to be responsible for the gametophytic male sterility of CMS-BT rice by transgenic experiments (Iwabuchi et al., 1993; Akagi et al., 1994; Wang et al., 2006b). Fertility restoration of CMS-BT rice is controlled by a nuclear gene, *Rf1*, which encodes a PPR-containing protein (Kazama and Toriyama, 2003; Akagi et al., 2004; Komori et al., 2004). Wang et al. reported that *Rf1* locus consists of two *Rf* genes, *Rf1a,* and *Rf1b* (Wang et al., 2006b). Both of the two *Rf1* genes can restore CMS-BT rice by silencing *orf79* transcript via different mechanisms respectively, endonucleolytic cleavage for RF1A and degradation of the dicistronic RNA for RF1B. However, when the two restorers are both present, the *Rf1a* gene has an epistatic effect over the *Rf1b* gene in the mRNA processing (Wang et al., 2006b). It has also been reported that the processed co-transcript of *B-atp6-orf79* is partially degraded, and the unprocessed *B-atp6-orf79* RNA possess the capability to translate. Thus, a certain amount of ORFH79 protein did accumulated in transgenic rice, however, the transgenic line did display a fertile phenotype (Kazama et al., 2008). This suggests a certain level of accumulation of ORF79 in rice does not result in CMS. The quantitative analysis of the amount of ORF79 protein that needs to accumulate to lead to pollen abortion remains to be researched.

## CMS-WA

Wild-Abortive CMS (WA-CMS) system is derived from the common wild species *Oryza rufipogon* Griff, which is applied most often for hybrid rice production (Lin and Yuan, 1980). This line shows a distinct difference both genetically and cytologically from that of CMS-BT and CMS-HL. Pollen abortion in CMS-WA occurs relatively earlier during microspore development, mainly at the uninucleate stage. It is sporophytically male-sterile, and the aborted pollen are amorphous. In contrast, the male sterility in both CMS-BT and CMS-HL rice is of the gametophytic type. The molecular mechanism of CMS-WA is poorly understood compared with that of CMS-HL and CMS-BT. Previously, an unedited 1.1 kb mitochondrial *orfB* gene transcript was found to be the candidate sterility gene of WA-CMS by RFLP (Das et al., 2010). But recently, Bentolila and Stefanov suggest *orf126* is the CMS-related candidate gene based on comparison of mitochondrial genomes by next-gen sequencing (Bentolila and Stefanov, 2012). The CMS and Rf genes of CMS-WA have still not been finally identified and cloned.

## CMS-HL

The CMS-HL line of rice was developed by the repeated backcrossing of a red-awned wild rice (*Oryza. rufipogon*) from Hainan Island, China with an early maturing indica variety called Lian-Tang-Zao. Hybrid rice varieties based on CMS-HL have been widely grown in China since the beginning of this century because it performs better agronomically and produces higher quality grain than those varieties based on other CMS systems (Liu et al., 2004). Compared with CMS-BT and CMS-WA, more systematic research has been undertaken to investigate CMS-HL rice. Wan and Li reported accumulation of high levels of ROS, significantly decreased adenylate content, as well as ATP/ADP ratio, and reduced mitochondrial membrane potential in Yuetai A compared with Yuetai B during microsporogenesis (Li et al., 2004; Wan et al., 2007). A similar phenomenon was also reported in other CMS systems such as *Brassica napus* and *Nicotiana tabacum* (Bergman et al., 2000; Teixeira et al., 2005). In fact, lower ATP levels were also found in their vegetative development, but only the pollen function is lost, suggesting that the mitochondrial activity is strictly required for gametophyte development during microsporogenesis in these lines.

In CMS-HL rice, a chimeric mitochondrial gene called *orfH79*, located downstream of *atp6*, has been proposed as the candidate gene causing the CMS trait of Honglian rice (Yi et al., 2002). Peng et al. confirmed the expression of *orfH79* result in the pollen abortion in HL-maintainer line using transgenic experiments (Peng et al., 2010). The nuclear Rf5 gene can restore the fertility of CMS-HL rice, moreover, a Gly-rich protein, GRP 162 were found to interact directly with RF5 to form a subunit of the restoration of fertility complex. This complex processes the CMS-associated transcript *atp6-orfH79*, which provides a new perspective on the molecular mechanism underlying fertility restoration (Hu et al., 2012).

To better understand the molecular mechanism of HL-CMS rice, proteomic analysis has been performed. Two-dimensional gel electrophoresis and isotope-code affinity tag (ICAT) technology have been used to analysis the protein expression profile between the Yuetai A and Yuetai B. Results showed that proteins associate with energy production were reduction in the anther of HL-CMS rice (Wen et al., 2007; Sun et al., 2009), suggesting a low level of energy production played an important role in inducing CMS-HL. Accumulation of ORFH79 in mitochondria impairs their normal function, proteomic analysis showed energy production protein were reduced in anther of CMS-HL rice, and physiological features showed the mitochondrial activity in CMS-HL rice was down-regulated. This inspired study of the mitochondrial complex proteome between Yuetai A and Yuetai B. BN-PAGE and mass spectrometry- based quantitative proteomics showed a reduced quantity of mitochondrial complexes in Yuetai A compared to Yuetai B, indicating a defect in mitochondrial complex assembly in the sterile line. This study gave us a new viewpoint on the mechanisms of CMS (Liu et al., 2012).

A series of CMS-associated mitochondrial genes in rice and other plants have been cloned and functionally studied, but how CMS proteins specifically cause pollen abortion remains unclear. A platform for studying mitochondria of rice reproductive tissue is the main obstacle to this study. More dexterous and sensitive systems need to be developed to study the mechanism of CMS in rice and other plants.

## EGMS in rice

Nongken 58S (NK58S), is the first spontaneous photoperiod-sensitive genic male-sterile (PGMS) mutant found in the japonica cultivar Nonken 58 (NK58) grown in Hubei Province, China in 1973. Large-scale application of two-line hybrid rice in agriculture was developed quickly with the discovery of NK58S (Shi, 1985; Shi and Deng, 1986). The fertility of NK58S is highly regulated by day length at specific inflorescence development stages. It retains complete male sterility when the day length (photoperiod) is longer than 14 h during the anther development, however, male fertility returns gradually when the day length is shorter than 14 h. In addition, the photoperiod-sensitivity can be affected by temperature: a high temperature slightly promotes complete male sterility under long-day conditions (He and Yuan, 1989). Peiai 64S (PA64S) is one such NK58S-derived line with an indica (*O. sativa* ssp. indica) genetic background. With wide compatibility and good agronomic traits, PA64S has become the most widely used female parent for two-line hybrid rice breeding. However, the fertility transition of PA64S is controlled mainly by temperature rather than by day length: PA64S exhibits male sterility at temperatures higher than 23.5°C during the anther development, but it converts to male fertility when the temperature is ~21–23°C. Long-day (14 h) conditions can suppress the degree of sterility to fertility conversion under low temperatures (21–23°C), but short-day (12 h) conditions cannot restore male fertility under high temperatures (Luo et al., 1992; Xu et al., 1999; Lu et al., 2007). Although two-line hybrids developed using this EGMS germplasm have made great achievements in improving rice yield in China during the past two decades, people knew less about the molecular mechanism of how the day length and temperature coordinately regulate the fertility transition of EGMS in rice.

Genetic analysis found that *pms3* located on chromosome 12 was the original mutation which converts Nongken 58 to become the PGMS rice NK58S. Recently, *pms3* was cloned and shown to encode a long non-coding RNA (lncRNA) named LDMAR. A sufficient amount of LDMAR is required for male fertility under long-day conditions. A spontaneous G-C mutation causing a SNP between NK58 and NK58S, eventually brings about heritable increased methylation in the promoter region of LDMAR, which reduces the level of LDMAR expression. This then results in premature programmed cell death (PCD) in anther development under long days, and hence male sterility (Ding et al., 2012a). In addition, Ding et al. reported that RNA-dependent DNA methylation (RdDM) is involved in the regulation of PGMS. Promoter siRNA of LDMAR derived from *AK11270* is associated with the DNA methylation level of LDMAR, which reduces the expression level of LDMAR, and therefore male sterility in Nonken 58S under long-day conditions (Ding et al., 2012b).

*P/TMS12-1,* which confers PGMS in the japonica rice line NK58S and TGMS in the indica rice line PA64S, encodes a unique non-coding RNA, which produces a 21-nucleotide small RNA named *osa-smR5864w*. This RNA shares identity with the product of *pms3* at the nucleotide level, which is responsible for the fertility of the pollen of NK58S and PA64S (Zhou et al., 2012). Taken together, these findings suggest that this non-coding small RNA gene is an important regulator of male development controlled by cross-talk between the genetic networks and the environmental conditions.

## The consensus of CMS, EGMS, and GMS

**Figure 1:**
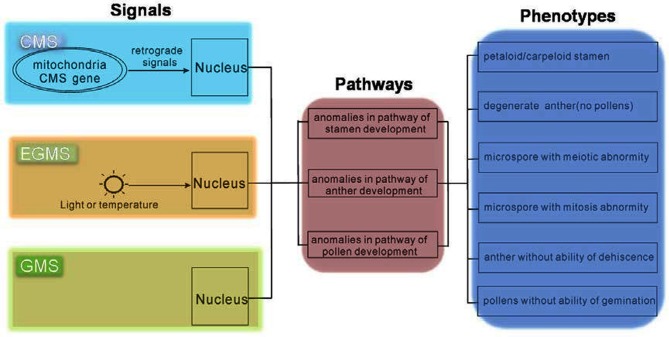
**Consensus in regulatory pathways of CMS, EGMS, and GMS.** Although the original signals of CMS, EGMS, and GMS are different, the pathways and phenotypes might be the same.

Mutants from various plant species allow us to use genetic strategies to uncover the developmental mechanisms of the male reproductive system (Wilson and Zhang, 2009; Borg and Twell, 2010; Twell, 2011). In Arabidopsis many important factors have been identified, and hence for this species, researchers have constructed relatively comprehensive pathways involved in the development of the male reproductive system (Wilson and Zhang, 2009; Twell, 2011). These Arabidopsis mutants were mainly produced by physical and chemical mutagens, such as X-rays, EMS, etc. Because the male sterility phenotypes of these mutants were caused by genomic mutation, we refer to this as GMS. Although differences in the reason for male sterility between these systems exist, CMS or EGMS and GMS might share a consensus in nucleus gene regulation (Figure 1).

In CMS plants, CMS-related proteins in mitochondria make retrograde signals to activate nuclear factors to direct downstream pathways defining pollen fate. For EGMS, the external environmental signals, light or temperature, are received by unknown receptors, and then transmit to the nucleus to guide pollen development. Therefore, as the phenotype of these two kinds of male sterility was both finally executed by the nucleus, the signals to cause male sterility in CMS and EGMS must impair the normal pathways that spatio-temporally regulate the development of the male reproductive system. In the mutants of male sterility (GMS), the normal pathways have been deteriorated by missing some key factors. From this perspective, the study of GMS occupies a core position for comprehensively understanding male sterility, including both CMS and EGMS.

**Figure 2:**
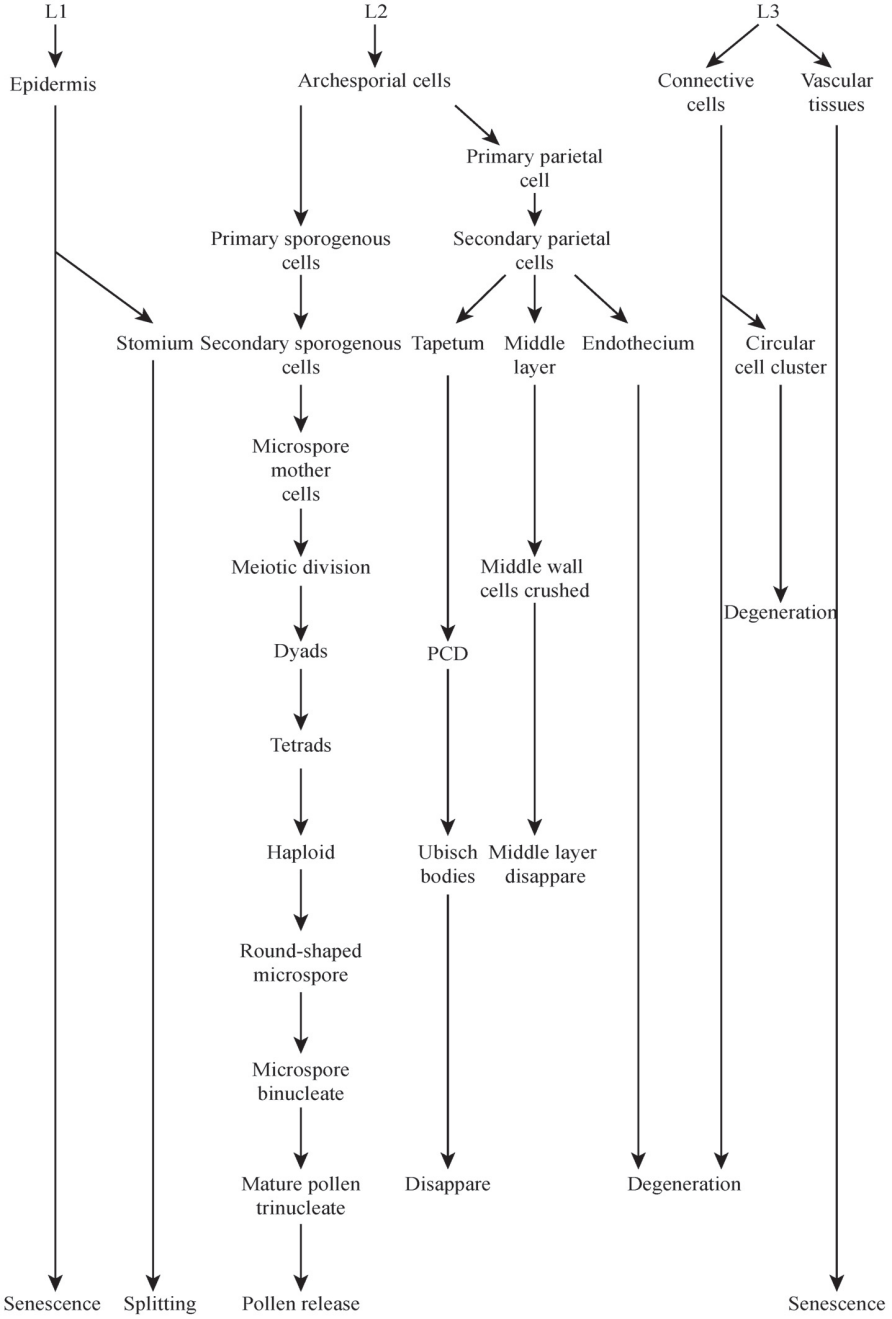
**Major events during rice microspores development and dehiscence.** Development stages and events were taken from Zhang et al. (2011). Some events were marked with boxes. L1, L2, L3 means layer 1, layer 2, and layer 3, respectively.

The development of the male reproductive system of rice is a very complex process (Figure 2) which includes the formation of the stamen with differentiated anther tissues, in which microspores/pollens are generated, which is followed by anther dehiscence and subsequently by pollination (Goldberg et al., 1993) (Figure 2 not show the stamen formation process).

## Network of male reproductive system development

**Figure 3:**
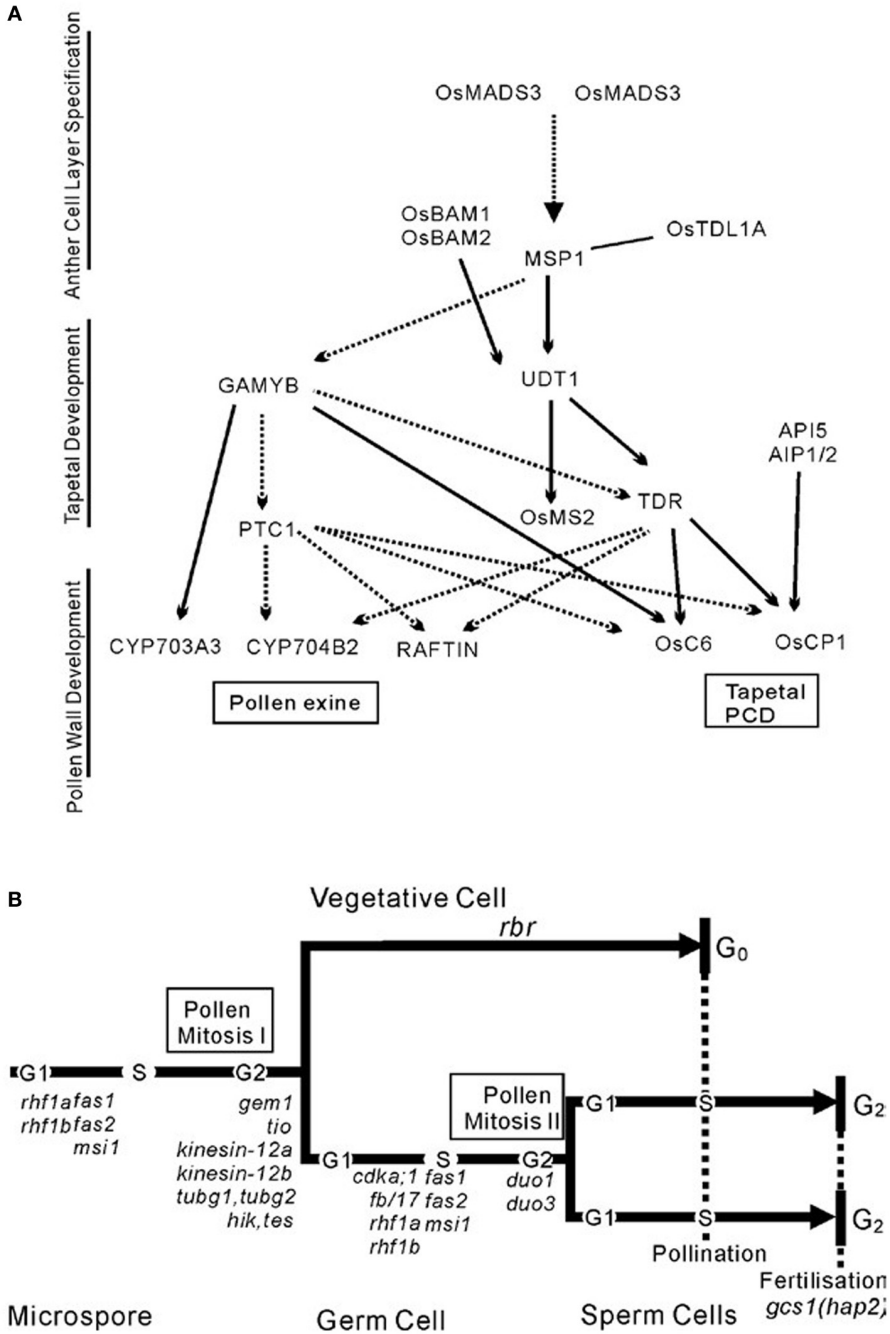
**Two local pathways of development of the male reproductive system. (A)** The networks of pollen development in rice. **(B)** Schematic illustrating the cell lineages and cell cycle progression in microspore development in the context of gametophytic mutations that affect asymmetric division, cell cycle progression, and patterning. This schematic is from Twell (2011).

Benefited from the identification of a number of male-sterile mutants in rice, a preliminarily regulatory network of pollen exine formation and tapetal development can be constructed (Figure 3A). *OsMADS3* and *OsMADS58* which belong to C-class MADS box transcription factor family were identified to control archesporial specification and the start of anther development (Yamaguchi et al., 2006). The phenotype of the MULTIPLE SPOROCYTE1 (*msp1*) mutant shows increased numbers of male and female sporocytes and disrupted anther wall layers. *MSP1* which encodes a LRR receptor-like kinase (LRR-RLKs) might play the function of initiating anther wall development and controlling male and female sporogenesis (Nonomura et al., 2003). *OsTPL1A* was found to bind *MSP1* to co-regulate sporogenesis (Zhao et al., 2008), and the interaction between them was identified by yeast two hybrid studies. BAM1 and BAM2, which also belong to the LRR-RLKs protein family, were found to act redundantly in defining the parietal cells that give rise to the endothecium, middle and tapetal cell layer, as these cell layers disappeared in the double mutant *bam1bam2* (Hord et al., 2006).* OsUDT1* which encodes a basic helix-loop-helix (bHLH) protein seems to be one of the earliest players in tapetal development (Jung et al., 2005). *OsTDR*, a possible regulator downstream of *OsUDT1*, controls tapetum development. It also encodes a bHLH protein and has been proposed as a trigger for rice tapetum PCD. The TAPETUM DEGENERATION RETARDATION (*tdr*) mutant has highly vacuolated and retarded PCD tapetum and degenerated microspores (Li et al., 2006). Another two genes, *OsCP1* and *Osc6* (Zhang et al., 2010a), which respectively encode a cysteine protease and a lipid transfer protein (*LTP*), were identified by chromatin immunoprecipitation-PCR and electrophoretic mobility shift assay (EMSA) as direct downstream targets of OsTDR protein. Recently, APOPTOSIS INHIBITOR5 (API5) and its two partners AIP1 and AIP2 were also found to bind the promoter of *OsCP1* and increase its expression level. They regulate tapetal PCD through regulating the expression of *OsCP1* (Li et al., 2011b). Through studying pollen exine, researchers have found *GAMYB* which is downstream of The PERSISTANT TAPETAL CELL1 (*PTC1*). The *GAMYB also* acts downstream of *MSP1-OsTDLIA* which is supported by the fact that down-regulation of *GAMYB* was observed in *msp1-4* anthers (Wang et al., 2006a). *PTC1* functions downstream of *GAMYB* (Aya et al., 2009) but on the same level as *TDR* in controlling anther and pollen development. Interestingly, the *TDR*, *OsCP1,* and *OsC6* that take charge of Tapetal PCD were found being regulated by *GAMYB* and *PTC1*, indirectly or directly. Meanwhile, the recently identified genes *CYP703A3*, *CYP704B2,* and *RAFTIN* that are involved in lipid metabolism and transport during pollen exine formation might act downstream of *GAMYB, PTC1,* and *TDR* (Li et al., 2010, 2011a). The data also show that *CYP704B2* and *RAFTIN* might be affected by *TDR* (Li et al., 2011a).

In contrast, the regulatory model to describe how cell cycle-associated genes control the process that diploid pollen mother cells undergo in meiosis and mitosis to produce mature pollen has also been preliminarily constructed in *Arabidopsis* (Figure 3B). Considering the high conservation of these genes, studies of their homologs in rice would probably lead to similar results, although many studies need to be performed in rice to confirm this.

## Engineered male sterility

Conventional breeding methods utilizing naturally occurring mutations to create male sterility have their shortcomings including: being time-consuming, the limitation of genotype numbers and the uncertainty of targets. However, we consider it would be possible to construct inducible male-sterile plants based on our design, because modern biotechnology has offered us new approaches to obtain male-sterile plants. Because of limitations in propagating male-sterile plants without segregations in the next generation or insufficient restoration of fertility for fruits or seed-setting of the hybrid varieties, only one engineered system is, so far, used in practical breeding. This exploration for engineering male sterility rice started as early as the 1990's when Ling et al. (1998) successfully developed completely sterile rice plants by co-transferring Barnase-ps1 and pHcintG genes into genomic DNA of the rice variety Tai-Pei-309.

Based on the target location of the transformed alien genes, the engineered male-sterile lines can be classified into two types: cytoplasmic male-sterile and nuclear male-sterile lines. Relative to the engineered nuclear male-sterile lines, development of engineered cytoplasmic male-sterile lines is more difficult due to the obstacle of transforming the target genes directly into mitochondrial or chloroplast genomes. Daniell et al. (2002) successfully introduced alien genes into the chloroplast genome, and obtained completely male-sterile plants with a novel method, and this method has been further successfully applied in *phaA* transformation research (Ruiz and Daniell, 2005). In this way, these researchers opened the door to breed environmentally friendly genetically modified rice sterile lines by preventing the diffusion of seeds or pollen grains containing alien genes into the environment.

Pollen development is controlled by a sequential expression of genes specially expressed in reproductive tissues, and is highly susceptible to the cellular environment. Any imbalance of the expression of genes related to anther and microspores development, such as starch synthesis, nutrition transportation, or energy production will lead to abnormalities and disable male gametogenesis. This provides the theoretical possibility of EGMS or GMS breeding. *PAIR1* (Nonomura et al., 2004) controls homologous chromosome pairing and cytokinesis in male and female meiocytes of rice. *WDA1* (Jung et al., 2006) shows a pivotal role in the biosynthesis of very-long-chain fatty acids in both outer and inner layers of anthers. Both of their mutants showed abortive pollens. In addition, the male-sterile phenotypes of mutants of *MSP1* (Nonomura et al., 2003), *MADS3* (Hu et al., 2011), *API5* (Li et al., 2011b), *UDT1* (Jung et al., 2005), and *WDA1* (Jung et al., 2006) which have been mentioned above given us evidences that plants would show male-sterile phenotypes if any of these genes were destroyed or silenced.

Theoretically, we could acquire male-sterile plants by disturbing the expression of any genes that were identified to take part in anther or pollen development. To date, very few of them have been successfully applied in practical hybrid rice breeding. Chen et al. (2007) used the *Ubiqutin* promoter to over-expressed UDP-glucose pyrophosphorylase 1 (*Ugp1*) which plays critical roles in rice growth, especially in pollen development. Interestingly, the attempt to over-express *Ugp1* actually silenced *Ugp1* and unexpectedly acquired a novel thermo-sensitive genetic male sterility rice. Pollen mother cells of *Ugp1*-silenced plants appeared normal before meiosis, but during meiosis normal callose deposition was disrupted. Consequently, the pollen mother cells began to degenerate at the early meiosis stage, eventually resulting in complete pollen collapse. This finding might give us a novel way to practical breeding of EGMS lines in rice using genes that are in the network of male reproductive development.

Engineered nuclear male sterility controlled by a single gene is easy to handle from the angle of using current molecular techniques, but a problem with these nuclear MALE-STERILE transformants is that they segregate for male fertility or sterility and must be over planted and rogued by hand or sprayed with herbicides to remove male-fertile plants (Ruiz and Daniell, 2005). This confines the application of engineered MALE-STERILE lines in a simple way for commercial production. Multiple gene controlled MALE-STERILE lines may help us avoid such insurmountable issues. Recently, Kubo et al. (2011) identified a novel sterility locus on rice chromosome 2 named Epistatic Factorfor S24 (EFS). EFS interacts with S24, a gene for indica and japonica hybrid male sterility, and shows a recessive sporophytic allele in japonica rice. When S24 is in combination with the homozygous japonica EFS allele (efs-j), male sterility will occur, while the indica allele (EFS-i) can dominantly counteract the pollen sterility caused by S24 heterozygosity. These results demonstrate that an additional epistatic locus is an essential element in the hybrid sterility caused by allelic interaction at a single locus in rice. If we combined these two epistatic loci with other phenotype markers, it will provide a possibility to employ allelic interaction of two loci in two different rice varieties to develop a robust GMS system for hybrid rice.

Alternatively, some novel male-sterile system created in other crops may be able to be applied in rice breeding practice. Zhang et al. (2010b) developed a novel system for creating male-sterile transgenic plants by down-regulation of the specifically expressed glyphosate tolerance CP4 EPSPS gene in male reproductive tissues. The CP4 EPSPS gene was driven by a modified CaMV 35S promoter with three tetracycline operator copies in the vicinity of the TATA box. The controllable transgenic plants could tolerate exposure of glyphosate but the male tissue was sensitive. The novel inducible male sterility system is applied and easy to handle, and it might be applicable to a wide range of crop plants including rice. Meanwhile, Engelke et al. (2010) constructed a male sterility and restoration system by interference with extracellular invertase activity in potato. Antisense repression of the anther-specific cell wall invertase or interference with invertase activity by expressing a protein inhibitor under the control of the anther-specific invertase promoter results in a block during early stages of pollen development, thus causing male sterility without any pleiotropic effects. Restoration of fertility was successfully achieved by substituting the down-regulated endogenous plant invertase activity by a yeast invertase fused to the N-terminal portion of potato-derived vacuolar protein proteinase II (PiII–ScSuc2), under control of the orthologous anther-specific invertase promoter *Nin88* from tobacco. The chimeric fusion PiII*–*ScSuc2 is known to be *N-glycosylated* and efficiently secreted from plant cells, leading to its apoplastic location. Furthermore, the *Nin88::PiII-ScSuc2* fusion does not show effects on pollen development in the wild-type background. Thus, such plants can be used as paternal parents of a hybrid variety, thereby the introgression of *Nin88::PiII-ScSuc2* to the hybrid is obtained and fertility is restored. Because of the conservative of plant invertases, this system could also be used in rice.

## Prospect

An increasing number of genes causing GMS have been identified by forward or reverse genetic strategies and can be integrated into some preliminary network systems (Figure 3). However, currently the in-depth study in this field to explore the network is confined by limited genetic materials. After all, the number of mutants is still relatively few compared to the number of genes in rice nuclear genome. Comprehensive resolution of the spatio-temporal profiles of key component of the male reproductive system at the transcriptomic and proteomic levels will greatly facilitate our understanding of the mechanisms of male reproductive development. Previous studies have accumulated large amount of transcriptomic (Becker et al., 2003; Lee and Lee, 2003; Honys and Twell, 2004; Pina et al., 2005; Borges et al., 2008; Haerizadeh et al., 2009; Tang et al., 2010; Wei et al., 2010; Hafidh et al., 2012) and proteomic (Mayfield et al., 2001; Chen et al., 2006; Dai et al., 2006, 2007; Sheoran et al., 2007; Pertl et al., 2009; Han et al., 2010; Lopez-Casado et al., 2012) data on development of pollen grains, but there are still a lot of blanks to fill in. For example, the proteomic study of post-translational modification, phosphorylation, and ubiquitination, etc., during the process is still limited (Mayank et al., 2012). In addition, epigenetic regulation by small non-coding RNAs and modification of DNA and histone has emerged for with roles in male reproductive system development (Johnson and Bender, 2009; Slotkin et al., 2009). However, with the great improvements in RNA and protein detection technology, such as single molecular sequencing (Fang et al., 2012) and label-free MS (Levin and Bahn, 2010), and combined with laser capture microdissection technology, we would have the ability to achieve more subtle spatio-temporal expression profiles with less tissue. In this way even sperm and vegetative cells that it was previously impossible to study with omic data, can become targets for studies.

Moreover, the study of how CMS and EGMS rice receive and transfer mitochondrial retrograde signals and light/temperature signals is another key and interesting aspect for male sterility. In HL-CMS, verifying the hypothesis that the level of ATP and ROS may play the retrograde signals from mitochondrion to nucleus and how the nucleus genes receive and transfer the signals needs further study. In PGMS, how the lncRNAs (pms3) sense the different photoperiod and alter their expression is also very interesting and awaits much deeper research.

Taken together, concerted efforts from multiple angles would pave the road to rapid progress in understanding the complex regulatory networks and finally enable us to attain a holistic concept for the development of rice male reproductive system. All the data harvested in these studies will definitely help us to freely manipulate fertility in rice and other crop plants to facilitate hybrid breeding in the future.

### Conflict of interest statement

The authors declare that the research was conducted in the absence of any commercial or financial relationships that could be construed as a potential conflict of interest.
